# Annotations

**Published:** 1890-10-25

**Authors:** 


### Annotations.

**Mixed Committees.**

Miss Louisa Twining in another column advocates the appointment of women on hospital committees. She points out that there are many matters connected with hospital economy which properly appertain to a woman's functions, and which would therefore be better done in all probability if women were to serve on the committees of management. She resents the opposition which some gentlemen display when a proposal to elect women governors is brought forward at the annual meetings. Theoretically we are inclined to agree with Miss Twining; but in practice it has been found that where ladies have been admitted to the management, the results have not justified the new departure. If it were possible to secure the appointment of women of business only, then no doubt nothing but good would result from the change. Unfortunately those who covet these positions are, as a rule, without experience, and, unfortunately, sometimes without judgment also. It used formerly to be a common practice to elect a ladies' committee, who were entrusted with certain details of the internal arrangements of some hospitals. These committees have, however, for the most part disappeared, chiefly because experience proved that they tended to introduce friction and discord rather than harmony and efficiency. No doubt where a hospital is fortunate enough to have amongst its governors a woman or women of the right kind, their election to a seat on the board of management would not only be expedient, but advantageous also. Unfortunately the right material is usually wanting, and so, we believe, many things must happen before Miss Twining's proposal wins popularity and secures wide recognition. The subject is, however, one well worthy of discussion, and we shall be glad to open our columns to those who take an interest in the subject, in the hope that by this means those best able to speak with authority will come forward and express their views and the reasons upon which they are based.

**Nurses and Balloonists.**

The height of absurdity was reached last week, when the members of the scientific society known as the "Balloon" held a meeting to discuss the grievances of hospital nurses. Alas! the trials of the hospital nurse, are, of the earth, earthy, and the members of this highly rarified society were all "in the air," as regarded their views of the question. They gazed from afar off at the subject, and their conclusions were strange. The paper of the evening was read by Mr. Nelson Hardy, and Mrs. Browne was present. Mr. Nelson Hardy, being two heroes rolled into one, is reported to have confessed he had never been attached to a general hospital, but still, as a member of the balloon society, given to sailing round the moon, he considered that nurses were overworked. Nurse Burn-Bailley seconded the resolution. Now this was rather mean of Nurse Burn-Bailley; we wanted to hear a real good ranting speech; others who knew nothing about the subject had talked at length, but courage failed that poor victim Nurse Burn-Bailley, and she merely "seconded the resolution." The Balloon Society ought to study the manner in which the Salvation Army makes its witnesses testify. Though this part of the proceedings was disappointing, the next speaker characterised the meeting as one of the most amusing entertainments he had ever attended. This high compliment was not appreciated, and Mr. Thomas Ryan (who happened to be attached to a general hospital and to have some practical knowledge of the subject in hand) was greeted with interruptions and cries of "Oh, oh!" The report goes on to say, "considerable confusion ensued": it generally does when a lot of people talk about things they don't understand: there might be confusion in a balloon if a nurse, learned in pharmacy and therapeutics, tried to govern the procedure of that gaseous body, and to throw out sandbags in opposition to the aeronaut. There were a few nurses and medical students present. We hope they felt properly humiliated by the very undignified proceedings. If there is dirty linen to be washed in the nursing world, let it at least be washed at home. Contempt and ridicule are bound to be the result of exposing such trivial grievances to a scoffing public. On the other side, if the Balloon Society is indeed a scientific organization, it had better not risk its reputation by giving other amusing entertainments on subjects of which it is profoundly ignorant. Has the world already forgotten Carlyle's phrase about "the eternal fitness of things"?

**Students in Pauper Hospitals.**

In last week's Hospital we gave a full report of the commencement of the Post Graduate Course at the Paddington Infirmary. It is our intention to report the proceedings from week to week, and we are proud to think that The Hospital has been the first journal to recognise and give full publicity and encouragement to the valuable scheme which Dr. Savill has done so much to foster and promote. It is not generally known that in the original draft of the Bill submitted to the House of Commons which created the Metropolitan Poor Law Infirmaries a clause was inserted throwing these institutions open for clinical instruction. For some reason or other, on the ground, we believe, that it was cruel to utilise the pauper when sick for clinical purposes, this beneficent clause was repealed in 1869, and so one great feature for good in Hardy's Act was defeated. We have had an opportunity of closely watching the effects of clinical teaching upon the patients in hospitals during the last twenty years, and we have no hesitation in stating that if we had to make our choice, when ill, between treatment in a clinical hospital, and treatment in an institution where no teaching was permitted, we should undoubtedly decide in favour of the former. Further, it cannot be denied that the absence of clinical teaching closes the poor-law infirmaries, and even renders the medical treatment in them far more perfunctory and unsatisfactory than it would otherwise be. No doctor can permit himself to carelessly examine any case in a clinical hospital, because the students are so observant and shrewd that such a practice must ultimately ruin the reputation of any one who pursued it. We are well aware that many of the medical officers attached to the poor-law infirmaries are highly-trained practitioners with a keen sense of duty, and that they labour assiduously to do the best for the patients entrusted to their care. Still no thoughtful person can doubt that to entrust two hundred and fifty or more beds to the sole charge of one medical man must necessitate an absence of thoroughness and attention which is not to the advantage of the patients. Despite the excellence of many of the poor-law infirmaries of the present day abuses exist in some of them which would make the ears of the public tingle did they know all the facts. Further, there are abuses connected with the infirmary wards of many workhouses in this country which are not only disgraceful but which the Local Government Board feel to be a reflection upon the whole system of poor-law medical relief. The Post Graduate class at the Paddington Infirmary is, therefore, to be welcomed and encouraged in every way. Should the movement spread the comfort and the improvement in the surroundings and treatment of the sick in our poor-law infirmaries must be great. Indeed, we believe the time is not far distant when the Legislature will insist upon the whole of these institutions being thrown open to clinical treatment. The sooner this reform is introduced the better for the interests of the sick, and of the public.

